# Impact of hydrogen concentrations on the impedance spectroscopic behavior of Pd-sensitized ZnO nanorods

**DOI:** 10.1186/1556-276X-8-68

**Published:** 2013-02-11

**Authors:** Muhammad Kashif, Md Eaqub Ali, Syed M Usman Ali, Uda Hashim, Sharifah Bee Abd Hamid

**Affiliations:** 1 Nano Biochip Research Group, Institute of Nano Electronic Engineering (INEE), Universiti Malaysia Perlis (UniMAP), Kangar, Perlis 01000, Malaysia; 2 Nanotechnology and Catalysis Research Centre, Universiti Malaya, Kuala Lumpur 50603, Malaysia; 3 Department of Electronic Engineering, NED University of Engineering and Technology, Karachi 75270, Pakistan

**Keywords:** Sol-gel, Pd-sensitized ZnO nanorods, Impedance behavior, Nyquist plot

## Abstract

ZnO nanorods were synthesized using a low-cost sol-gel spin coating technique. The synthesized nanorods were consisted of hexagonal phase having *c*-axis orientation. SEM images reflected perpendicular ZnO nanorods forming bridging network in some areas. The impact of different hydrogen concentrations on the Pd-sensitized ZnO nanorods was investigated using an impedance spectroscopy (IS). The grain boundary resistance (*R*_gb_) significantly contributed to the sensing properties of hydrogen gas. The boundary resistance was decreased from 11.95 to 3.765 kΩ when the hydrogen concentration was increased from 40 to 360 ppm. IS gain curve showed a gain of 6.5 for 360 ppm of hydrogen at room temperature. Nyquist plot showed reduction in real part of impedance at low frequencies on exposure to different concentrations of hydrogen. Circuit equivalency was investigated by placing capacitors and resistors to identify the conduction mechanism according to complex impedance Nyquist plot. Variations in nanorod resistance and capacitance in response to the introduction of various concentrations of hydrogen gas were obtained from the alternating current impedance spectra.

## Background

While hydrogen gas has been increasingly used as a clean and green fuel in household and transportation appliances, the absence of color, odor, and taste has made it difficult to trace and detect hydrogen under complex matrices [1]. Hydrogen is a light and diffusible gas (diffusion coefficient of 0.61 cm^2^/s in air) [1] with a wide ranging inflammability (4% to 75%) [2]. Even 4.65% hydrogen in air is sufficient to cause explosion [2]. Thus, the detection and leakage control of this gas is a challenging task, and there is an increasing demand in the development of methodology for the ultrasensitive detection of hydrogen.

Previously, selective H_2_ sensors were proposed for the detection of hydrogen leakage in solid-state fuel cells [3], proton exchange membrane fuel cells [3], hydrogen engines [4], and hydrogen storage devices [5]. Bamsaoud et al. [6] used nanoparticulate tin oxide (SnO_2_)-based resistive films for the selective detection of hydrogen against relative humidity and CO_2_ at 265°C. Wang et al. [7] used mesostructured SnO_2_ for the selective detection of hydrogen against methane, butane, and CO at 300°C. Tianshu et al. [8] studied the effect of different Cd-doped SnO_2_-based sensors from 200°C to 450°C and selectively detected 1,000 ppm of hydrogen against 1,000 ppm of CO and 1,000 ppm of isobutane (i-C_4_H_10_) in the absence of ethanol vapor at a Cd to Sn ratio of 0.1. Lupan et al. [9] detected 10% H_2_ in N_2_ at 112°C using nanosensor based on zinc oxide (ZnO) nanorods. Garcia et al. [10] utilized Pd-decorated ZnO and tungsten oxide (WO_3_) nanowires for the selective detection of 4,500 ppmv H_2_/N_2_ at 100°C. Yamazoe et al. [11] studied the effect of different additives on SnO_2_ films and found that Ag-SnO_2_ film showed the highest sensitivity and selectively towards 0.8% hydrogen against 0.5% CH_4_, 0.2% C_3_H_8_, and 0.02% CO. Choi et al. [12] used electrospun Pd-doped SnO_2_ hollow nanofibers for the detection of hydrogen under ethanol background. Lupan et al. [13] studied the hydrogen selective response at room temperature using tetrapod ZnO sensor. Using an UV source of activation, they detected 100 ppm of hydrogen against 100 ppm of CO, isobutane, CH_4_, CO_2_, and SO_2_. However, in the forthgoing era, there is a requirement of hydrogen sensors having superior stability, sensitivity, and fast response time, along with low operating power and weight.

Recently, semiconductor metal oxides have been increasingly used in humidity, gas, and chemical sensing devices [14]. This is probably because of their simple fabrication, low cost, size reduction, appreciable sensitivity, and fast response time [1]. Catalytic metal-doped semiconductor metal oxides such as SnO_2_[15], titanium dioxide (TiO_2_) [16], ZnO [17], and WO_3_[18] have been used to develop hydrogen sensors. The addition of suitable quantity of appropriate metal catalyst enhances chemical reaction through the lowering of activation energy at the metal oxide thin film and target gas interfaces. The addition of metal as a catalyst also improves target response and selectivity at room temperature [19]. ZnO nanorods and nanowires are particularly promising for these applications because of its large surface area, wide bandgap and exciton energy, fascinating sensitivity, biocompatibility, low weight, and resistance to rust formation [20]. For hydrogen sensing applications, surface modifications of ZnO with metal additives such as Pt, Pd, and/or Au through various techniques have been under intensive investigations [19,21,22]. Several studies have demonstrated that Pd doping on ZnO nanowires and nanorods enhances room temperature hydrogen sensing through the catalytic dissociation of molecular hydrogen to atomic hydrogen at room temperature [21]. The predominant methods documented to synthesize ZnO nanorods for this particular application are chemical vapor deposition (CVD) and molecular beam epitaxy (MBE) [21,22]. However, both CVD and MBE methods involve high temperature growth and expensive instrumentations which are not available and affordable in ordinary laboratories. These techniques also need gold (Au) and/or other expensive metal coatings for the synthesis of ZnO nanorods and nanowires [10,11]. Moreover, Pd doping on the synthesized zinc oxides requires RF sputtering which also demands expensive laboratory setup. Additionally, previous researchers used DC measurements [19,21,22] which cannot elucidate the contributing factors such as the grain, grain boundary, and electrodes that might influence the target response on the Pd-sensitized ZnO nanostructures.

Recently, sol-gel spin coating technique has received enormous attention because of its simplicity, affordable instrumentations, low cost, and controllable growth temperatures [23]. In this paper, *c*-axis-aligned hexagonal ZnO nanorods with good crystalline properties were synthesized using a low-cost spin coating technique. Pd doping on the synthesized ZnO was performed using very simple instrumentations that require only micropipette and hot plate. However, to the best of our knowledge, such a method is not documented for the synthesis of Pd-sensitized ZnO nanorods for hydrogen detection applications. Room temperature hydrogen sensing was performed in a low-cost homemade gas chamber, and superior sensitivity and stability over the literature-reported Pd-sensitized ZnO nanorods were achieved. Potential contributors to sensor functionalities were elucidated through impedance study which is an AC measurement technique that can define contributions from grain, grain boundary, electrodes, and other associated elements. The simplicity and reproducibility of the method suggested its potential applications in the large-scale synthesis of Pd-sensitized ZnO nanorods for use in hydrogen, chemical, and other gas sensing devices that involved Pd-mediated catalysis.

## Methods

**Figure 1:**
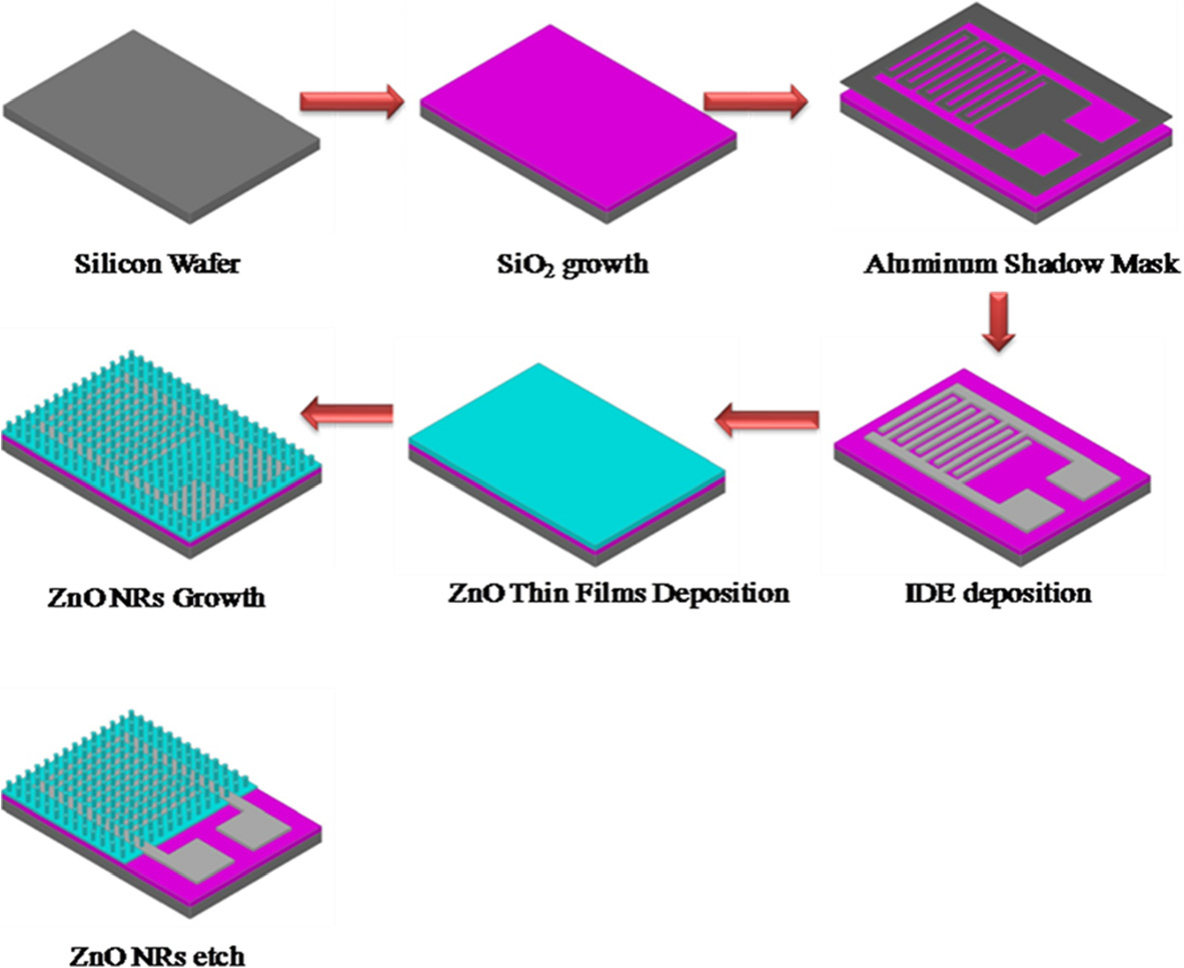
Process flow for the fabrication of ZnO nanorods device.

ZnO nanorods were synthesized on silicon dioxide substrate as described in our previous research [24]. Briefly, zinc acetate dihydrate (98%; Sigma-Aldrich Corporation, St. Louis, MO, USA) was mixed in 2-methoxyethanol (99.8%; Sigma-Aldrich) where the molarity of Zn was maintained at 0.2 M. After 30 min of stirring at room temperature, the hot plate temperature was ramped up to 60°C. Monoethanolamine (MEA) (99%; Merck & Co., Inc., Whitehouse Station, NJ, USA) was added dropwise as a stabilizer under constant stirring. The molar ratio of MEA/Zn was maintained at 1:1. The stirring was continued until the solution turned into transparent from its initial whitish appearance. The prepared solution was aged for 24 h. The process flow for the device fabrication is depicted in Figure 1.

An oxide layer of approximately 1-μm thickness was grown on a p-type silicon substrate of resistivity 1 to 50 Ω cm through a wet oxidation process. Prior to the oxide growth, the wafer was cleaned with RCA1 and RCA2 solutions followed by draining in dilute HF to remove the native oxide. An interdigitated electrode layer was deposited onto the oxide layer through Cr/Au evaporation using a hard mask and Auto 306 thermal evaporator (Edwards High Vacuum International, Wilmington, MA, USA). ZnO seed layer was deposited on the thermally oxidized silicon substrate using a spin coater rotating at 1,000 rpm for 10 s and then ramped up to 3,000 rpm for 45 s. After coating the seed layer, the film was dried at 250°C for 20 min. The coating and drying processes were repeated five times. After depositing five successive layers, the sample was incubated in a furnace to anneal the thin film at 450°C for 1 h under air atmosphere.

For the growth of ZnO nanorods, the prepared substrate was inserted inside a Teflon sample holder at the cut edges to keep the deposited side downward inside the growth solution. The growth solution was prepared by mixing zinc nitrate hexahydrate (99%; Sigma-Aldrich) and hexamethyltetramine (99%; Merck) in deionized (DI) water, and the final concentration of the solution was maintained at 25 mM. The beaker was placed inside a preheated oven, and the growth process was continued at 90°C for 6 h. The prepared ZnO nanorods were washed in IPA and DI water to remove the excess and contaminated salts. Subsequently, the synthesized ZnO nanorods were annealed at 450°C for 1 h under air environment. For Pd doping, 0.01 M solution of Pd was prepared by mixing the required amount of palladium chloride (PdCl_2_, 99.999%; Sigma-Aldrich) in ethanol. The solution was stirred overnight to completely dissolve the Pd particles. Five-microliter portion of the above solution was precisely transferred onto the synthesized ZnO nanorods using a micropipette, and the whole mixture was heated at 250°C for 5 min to dry out the residual chloride.

The structural properties of the Pd-sensitized ZnO nanorods were investigated using Bruker X-ray diffractometer (D8 Advance, Bruker AXS GMBH, Karlsruhe, Germany) with Cu Kα radiation at *λ* = 1.5406 Å. The X-ray diffraction (XRD) pattern was recorded in the range of 20° to 60° operating at a voltage of 40 kV and a current of 40 mA. The X-ray spectra peak analysis was carried out by Diffraction plus 2003 version of Eva 9.0 rev.0 software. The material composition was analyzed using X-ray photoelectron spectroscopy (XPS) (Omicron Dar400, Omicron, Erlangen, Germany). The chamber pressure was maintained at 2.4 e−10 Torr throughout the measurement. CasaXPS software was used for the XPS peak deconvolution. Morphological studies were performed using a scanning electron microscope (JEOL JSM-6460LA, Akishima, Tokyo, Japan). Gas sensing measurements were carried out in a homemade gas chamber of 3-L capacity. The base of the chamber was made up of stainless steel, and the upper area was covered with a high-vacuum glass dome. All the measurements were performed under atmospheric pressure. The chamber inlet was connected with the air pump and 1% H_2_ in balance N_2_ gas (Moxlinde, Malaysia). The flow of 1% H_2_ gas was regulated using a mass flow controller (GFC-17, 0 to 100 ml/min; AALBORG, Orangeburg, NY, USA), whereas the air flow was controlled using a mass flow meter. Impedance spectra were collected at room temperature (RT) in the frequency range of 1 Hz to 10 MHz using Novocontrol alpha high-frequency analyzer (Hundsangen, Germany) under the exposure of variable ppm levels of hydrogen.

## Results and discussion

**Figure 2:**
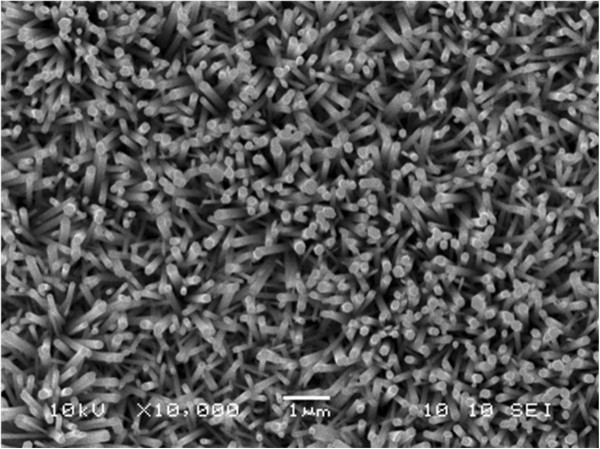
SEM image of Pd-sensitized ZnO nanorods.

The scanning electron micrograph depicting the morphological feature of ZnO nanorods grown on a thermally oxidized silicon substrate is shown in Figure 2. Uniformly distributed perpendicular and oblique ZnO nanorods of hexagonal shape having 50- to 100-nm diameter and 2- to 3-μm length were observed.

**Figure 3:**
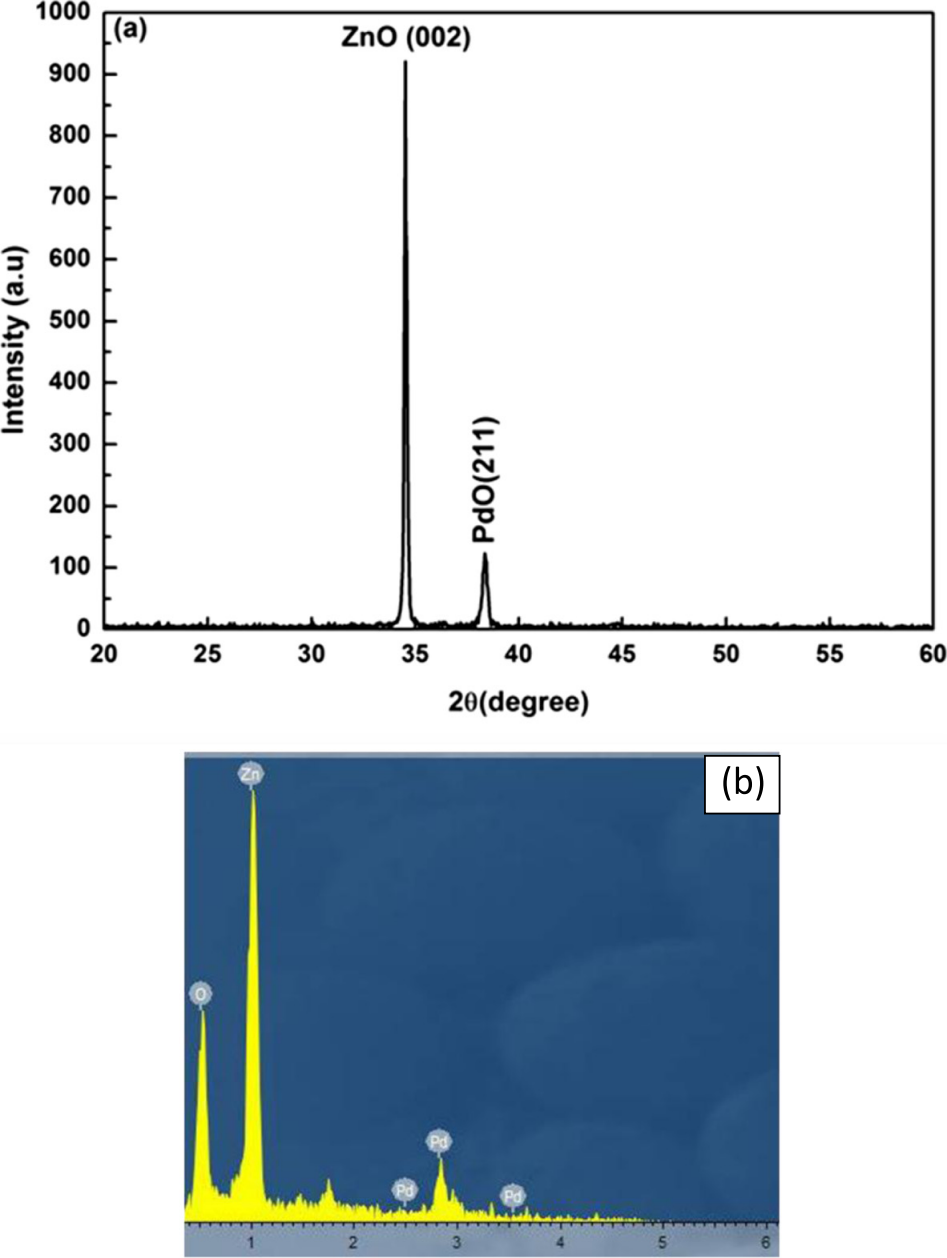
XRD (a) and EDS (b) spectra of Pd-sensitized ZnO nanorods.

The XRD spectra demonstrated two noticeable peaks at 34.5° (002) and 38.53° (211) planes (Figure 3a). The sharp peak located at 34.5° (002) plane of the synthesized ZnO nanorods revealed their high-quality crystals and *c*-axis alignment. The second peak at 38.53° (211) plane confirmed the presence of palladium oxide (PdO). The EDX spectrum of Pd-sensitized ZnO nanorods is presented in Figure 3b. The EDX spectrum demonstrated the presence of zinc, oxygen, and palladium, which indicated the absence of any other impurities in the prepared sample.

**Figure 4:**
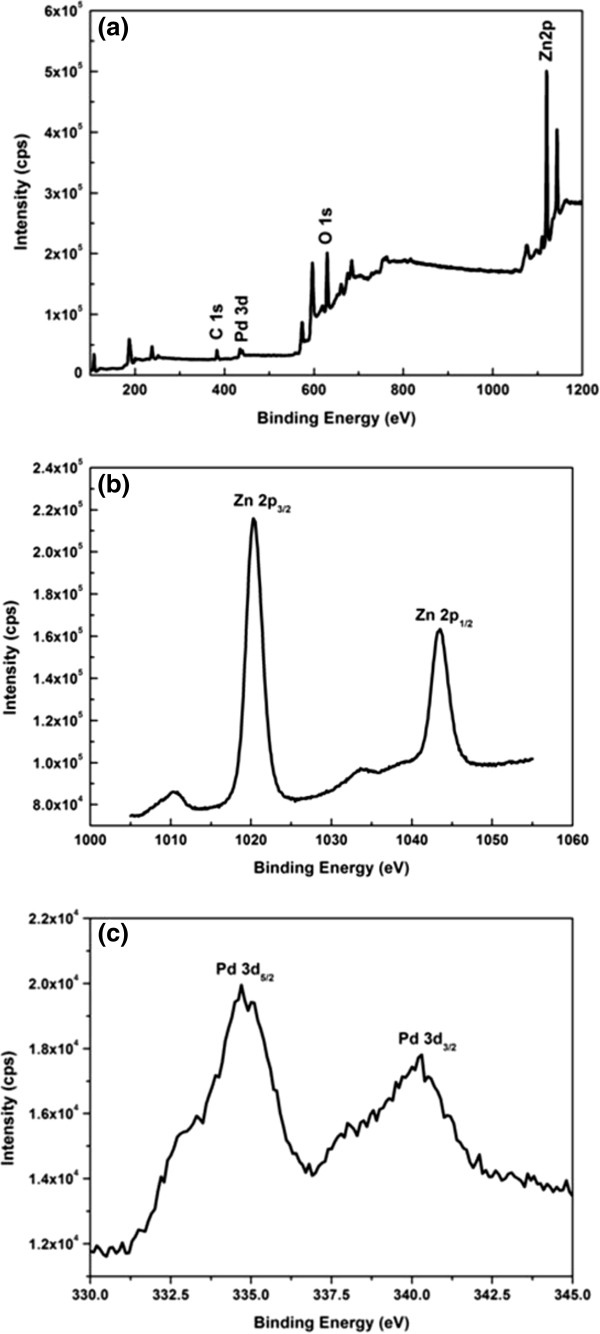
**XPS spectra of Pd-sensitized ZnO nanorods.** (**a**) Survey spectra, (**b**) Zn 2*p* spectra, and (**c**) the deconvolution spectra in Pd 3*d* region.

The surface composition of Pd-sensitized ZnO nanorods was further investigated using an XPS spectroscopy (Figure 4a) which reflected the presence of Zn, O, Pd, and carbon. The carbon peaks were due to the unavoidable air exposure during inserting the sample in an XPS chamber [25]. The peaks appearing at 284 and 288 eV were due to C-O and C=O bonds [26]. No other contaminants were detected on the Pd-sensitized ZnO nanorod surfaces. The XPS spectra of ZnO and PdO regions of our samples can be seen in Figure 4b,c. The Pd-sensitized ZnO nanorods showed two peaks at 1,020 and 1,043 eV that correspond to the distribution of Zn 2*p*_3/2_ and 2*p*_1/2_ core levels [25]. The binding energy peak for Pd 3*d*_3/2_ and Pd 3*d*_5/2_ core levels were observed at 340.82 and 334.7 eV, reflecting the presence of doped Pd in the form of PdO in the Pd-sensitized ZnO nanorods.

**Figure 5:**
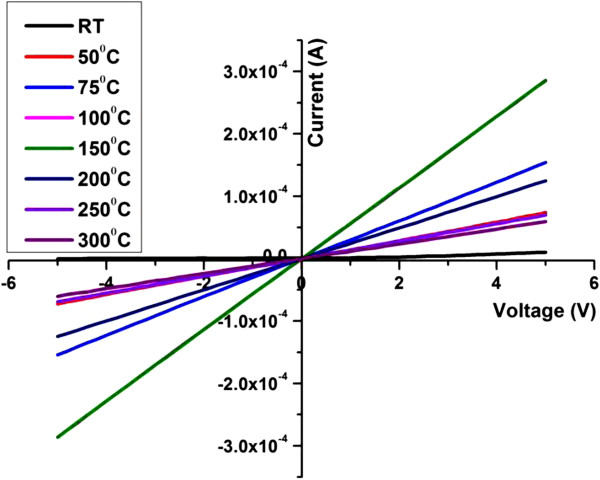
***I*-*V *curves of Pd-sensitized ZnO nanorods from RT to 300°C.**

The ohmic behavior was studied to understand the operational stability of the fabricated device. The current to voltage (*I*-*V*) characterization curve of the Pd-sensitized ZnO nanorods is depicted in Figure 5. It can be observed that the device exhibited a linear relation between the current and voltage. The *I*-*V* curve revealed the enhancement in current from room temperature to 200°C. Further increment in temperature (200°C to 300°C) resulted in the decrement of current flow. The current increment indicated that the electrons gain sufficient energy to overcome the barrier height between the grains with increasing temperature. The decrement in current value above 200°C was due to the formation of chemisorption region at elevated temperatures (200°C ~ 500°C) [27,28] where oxygen molecules adsorbed on the surface of metal oxide trapping electrons. In low temperature range, oxygen molecules were mainly physically adsorbed on the surface. However, at high operating temperature, the absorbed oxygen accepts free electrons from the conduction band of ZnO and be converted into oxygen ions (O^2−^ and O^−^). These oxygen ions (O^2−^ and O^−^) increase the surface resistance of the ZnO nanorods. In high temperature range, the adsorbed oxygen molecules turn into chemisorptions (i.e., chemical bond attractions), and the concentration of the adsorbed oxygen molecules on the surface gradually raise. As a result, the absorbed oxygen could trap more free electrons from the conduction band of ZnO to be converted into oxygen ions (O^2−^ and O^−^), resulting in an increase in surface resistance of the ZnO nanorods. In other words, when the oxygen molecules from the atmosphere are chemisorbed, it attracts the electrons from the conduction band causing a bending in band that creates a surface barrier and electron depletion or space charge layer. These lead to a reduction in conductivity and increase in resistivity in the metal oxide surface. Thus, the band edge bending in the conduction and valence band was related to the change in surface charge distribution.

Alternating current (AC) impedance spectroscopy was used to investigate the sensing mechanism in which the potential contributors could be defined [29]. Generally, the conduction process (*R*) and polarization behavior (*C*) become dominant in sensing mechanism. The device microstructures are composed of grains, grain boundaries, and the metal/ZnO contact. In the Nyquist plot, the major role players in the high, intermediate, and low frequencies are grains (bulk), grain boundaries (*R*_gb_, *C*_gb_) and the metal-semiconductor contact (*R*_c_, *C*_c_) [30]. In order to achieve a single semicircle from the prescribed components, the time constant *τ* associated with these components must be identical [31]:

(1) $$\tau =R_{\text{g}}C_{\text{g}}+R_{\mathrm{gb}}+R_{\text{c}}C_{\text{c}}.$$

**Figure 6:**
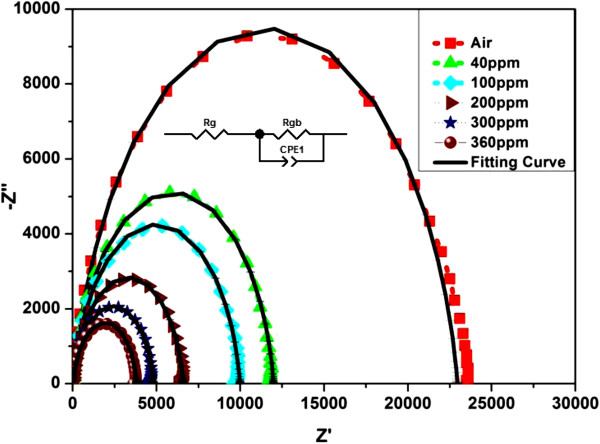
**Nyquist plot of Pd-sensitized ZnO nanorods as a function of different H**_**2 **_**concentrations at room temperature.**

The total impedance *Z*_T_ of the device structure can be drawn as follows:

(2) $$Z_{\text{T}}=Z_{\text{g}}+Z_{\mathrm{gb}}+Z_{\text{c}},$$

where *Z*_g_, *Z*_gb_, and *Z*_c_ represent the complex impedance contribution of the grains, grain boundaries, and the electrode contacts, respectively [32]. The grain resistance can be estimated from the interception of the arc at high frequency with the real axis [32]. Every individual semicircles has its own unique relaxation frequency *ω*_max_ (the frequency at the top of the arc), which can be represented as *ω*_max_*RC* = *ω*_max_*τ* = 1, where *R* and *C* represent the resistance and capacitance of the equivalent circuit and *τ* represents the relaxation time that depends only on the intrinsic properties of the material [33]. The effect of hydrogen gas on the impedance behavior of the sensor at different concentrations is shown in Figure 6.

It was observed that when the gas concentration gradually increased from 40 to 360 ppm, the diameter of the arc decreased. The Z^′′^ maximum values were smaller than the half values of the Z^′^ maximum, demonstrating the contribution from the constant phase elements (CPEs) in the equivalent circuit [29]. The best-fitted value for capacitance was obtained by replacing *C* with a CPE, which frequently describe the behavior of polycrystalline materials having inhomogeneous microstructures such as the grain boundary that gives rise to different distributions of respective relaxation time. The impedance of a CPE was clearly described in [34].

(3) $$Z_{\mathrm{CPE}}=\frac{1}{A{\left(\mathrm{j\omega}\right)}^{p}},$$

where *A* is a constant and *p* is a dimensionless parameter with value of less than unity. When *p* = 1, the equation represents the characteristics of a capacitor with *A* = *C*. The values noted in Table 1 shows that the resistance *R*_gb_ was varied because of the flow of different hydrogen concentrations. When the ZnO nanorods are exposed to air, oxygen molecules can capture free electrons from ZnO nanorods and form a surface depletion layer, which reduces the conducting width of ZnO nanorods and increases the potential barrier of the contacts between the ZnO nanorods. The surface depletion layer controls the density and mobility of electrons in the ZnO nanorods. When the ZnO nanorods are exposed to hydrogen, the adsorbed oxygen releases the previously trapped electrons back to the conduction band. The depletion width decreases as a result of the decrease in surface oxygen. This results in an increase in electron concentration of ZnO nanorods and a decrease in height of the barrier potential at the grain-grain contacts. Thus, the impedance of the ZnO nanorods decreases as the hydrogen concentration increases. Thus, it could be concluded that the hydrogen concentration significantly affects the grain boundary resistance which facilitates its detection.

**Table 1:** **Modeled RC parameters for Pd-sensitized ZnO nanorods under different H**_**2 **_**concentrations at room temperature**

**H**_**2 **_**(ppm)**	***R***_**gb **_**(Ω)**	***C***_**PE **_**(nF)**	***p *value**
0	22,938	4.99	0.89
40	11,950	3.53	0.9
100	9,950	3.5	0.9
200	6,550	2.938	0.91
300	4,780	2.88	0.91
360	3,765	2.83	0.91

However, the variation in the capacitance values was not significant. This reflected that the hydrogen gas mainly affects the surface charge region of the grain boundaries of Pd-sensitized ZnO nanorods.

**Figure 7:**
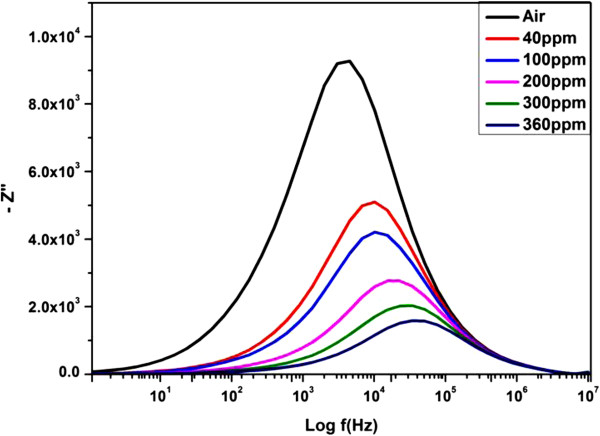
**Imaginary parts of impedance for Pd-sensitized ZnO nanorods under different H**_**2 **_**concentrations at room temperature.**

The peak frequencies related to the relaxation frequencies of the impedance were also estimated by plotting the −Z^′′^ versus the logarithmic frequency curve (Figure 7). It was observed that the imaginary part of impedance decreased as the gas concentration increased [2]. The decrement in the impedance imaginary part was related to the carrier concentrations. As the hydrogen concentration increases, the barrier height decreases causing more carriers to flow. This results in a decrease in impedance. It was also observed that the peak frequency shifted toward higher frequencies with increasing hydrogen concentration. The shifting of the peak towards high frequencies is related to an ease in the flow of charge carriers to the AC electric field [35]. The broadening of peak with an increase in hydrogen concentration was due to the different distribution of relaxation time [33,36]. The relaxation process may be due to the presence of electrons and/or immobile species [33].

**Figure 8:**
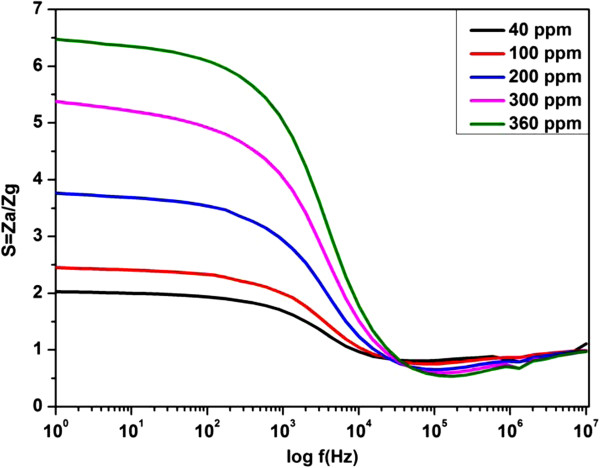
**Gain curve for Pd-sensitized ZnO nanorods as a function of different H**_**2 **_**concentrations at room temperature.**

The sensitivity of the fabricated ZnO nanorod sensor was evaluated as a function of frequency and hydrogen concentration using the equation given below:

(4) $$S=\frac{Z_{\text{a}}}{Z_{\text{g}}},$$

where *Z*_a_ represents the impedance of air and *Z*_g_ represents the real part of impedance under hydrogen flow. Figure 8 displayed the effect of frequency at different parts per million (ppm) values of hydrogen on Pd-sensitized ZnO nanorods at room temperature. The sensitivity of our device at room temperature was better than the reported literature values at 400°C [2]. The noticeable change in the sensitivity was observed in the frequency range of 1 Hz to 100 kHz.

In 1 Hz- to 100-kHz range, the space charge region rules the conductivity process. There is a sharp decrement in the sensitivity with the increment of frequency and little variation in the gain values at frequency higher than 100 kHz, where the conductivity is mainly dependent on the surface charge of the grains. This revealed that a suitable selection of frequency could achieve maximum gain in sensitivity.

The sensing mechanism can be described from the following aspects: The oxygen molecules from the ambient atmosphere were initially adsorbed onto the ZnO surface. The electrons were extracted from the conduction band of the ZnO material and were converted to a single or a double oxygen ion and became ionosorbed on the surface [2]. This led to a decrease in electron concentration and consequently an increase in resistance. This mechanism can be described as follows [2,37]:

(5) $${\text{O}}_{2}\left(\text{g}\right)+e^{-}\to {\text{O}}_{2}^{-}\left(\text{ads}\right).$$

The reaction of the hydrogen or any reduction gases with the ionosorbed ${\text{O}}_{\text{ads}}^{-}O$ results in the release of the captured electrons back to the conduction band. This results in an increase in electron concentration, decreasing the resistance which could be explained by the following reaction [2]:

(6) $${\text{H}}_{2}{\text{O}}_{\text{ads}}^{-}\to {\text{H}}_{2}\text{O}\left(\text{g}\right)+e^{-}.$$

When the hydrogen is introduced, PdO is reduced to metallic palladium, returning electrons to ZnO. Hydrogen molecules adsorbed on palladium simultaneously spill over the surface of ZnO, activating the reaction between hydrogen and the adsorbed oxygen:

(7) $$\text{PdO}+{\text{H}}_{2}\to \mathrm{Pd}+{\text{H}}_{2}\text{O}.$$

At elevated temperature, Pd is oxidized by the chemisorbed oxygen:

(8) $$2\mathrm{Pd}+2O_{\text{abs}}\to 2\text{PdO}.$$

The weak bonding of Pd atoms with the oxygen gas results in the dissociation of the complex at relatively low temperature releasing atomic oxygen. The oxygen atoms migrate along the surface of the grains. This migration is induced by the Pd catalyst and is known as spillover of the gaseous ions [38]. Thus, the oxygen atoms capture electrons from the surface layer forming an acceptor surface at the grain boundary. The presence of catalyst atoms activates the reaction between reducing gases and the adsorbed oxygen [39-41]. Thus, the Pd sensitization on the ZnO nanorod surface enabled the hydrogen sensing at relatively low operating temperature.

## Conclusions

A hydrogen sensor was successfully developed using Pd-sensitized ZnO nanorods synthesized on oxidized silicon substrate using a sol-gel spin coating technique. The sensor detected ppm level hydrogen at room temperature with more sensitivity over the literature-reported values for the ZnO-based sensors. The variation in the resistance value of the grain boundary which was the basis of analyte detection mechanism was due to the sole variation in hydrogen concentration. Nyquist plot strongly supported the impedance findings.

## Competing interests

The authors declare that they have no competing interests.

## Authors’ contributions

The work presented here was performed in collaboration of all authors. MK carried out the fabrication and electrical characterization of Pd-sensitized ZnO nanorods and drafted the manuscript. MEA and SMUA proofread the manuscript and corrected the language. UH supervised the work. SBAH provides the lab facilities for the XRD measurements. All authors read and approved the final manuscript.
